# Efficacy of a coxsackievirus A6 vaccine candidate in an actively immunized mouse model

**DOI:** 10.1080/22221751.2021.1906755

**Published:** 2021-04-21

**Authors:** Sha-Sha Qian, Zhen-Ni Wei, Wei-Ping Jin, Jie Wu, Yan-Ping Zhou, Sheng-Li Meng, Jing Guo, Ze-Jun Wang, Shuo Shen

**Affiliations:** Wuhan Institute of Biological Products Co. Ltd., Wuhan, People’s Republic of China

**Keywords:** CV-A6 vaccine, efficacy, active immunization-challenge, mouse model, HFMD

## Abstract

Coxsackievirus A6 (CV-A6) has been emerging as a major pathogen of hand, foot and mouth disease (HFMD). Study on the pathogenesis of CV-A6 infection and development of vaccines is hindered by a lack of appropriate animal models. Here, we report an actively immunized-challenged mouse model to evaluate the efficacy of a Vero-cell-based, inactivated CV-A6 vaccine candidate. The neonatal Kunming mice were inoculated with a purified, formaldehyde-inactivated CV-A6 vaccine on days 3 and 9, followed by challenging on day 14 with a naturally selected virulent strain at a lethal dose. Within 14 days postchallenge, all mice in the immunized groups survived, while 100% of the Alum-only inoculated mice died. Neutralizing antibodies (NtAbs) were detected in the serum of immunized suckling mice, and the NtAb levels correlated with the survival rate of the challenged mice. The virus loads in organs were reduced, and pathological changes and viral protein expression were weak in the immunized mice compared with those in Alum-only inoculated control mice. Elevated levels of interleukin-4, 6, interferon γ and tumour necrosis factor α were also observed in Alum-only control mice compared with immunized mice. Importantly, the virulent CV-A6 challenge strain was selected quickly and conveniently from a RD cell virus stock characterized with the natural multi-genotypes. The virulent determinants were mapped to V124M and I242 V at VP1. Together, our results indicated that this actively immunized mouse model is invaluable for future studies to develop multivalent vaccines containing the major component of CV-A6 against HFMD.

## Introduction

Hand, foot and mouth disease (HFMD) is a highly contagious disease that affects infants and children around the world [1,2]. Enterovirus A71 (EV-A71), coxsackievirus A16 (CV-A16), coxsackievirus A10 (CV-A10) and coxsackievirus A6 (CV-A6) are identified as the primary HFMD-related pathogens, belonging to species A in the genus *Enterovirus* within the family *Picornaviridae* [3].

The prototype strain of CV-A6, Gdula (GenBank ID AY421764) was isolated in the USA in 1949 [4]. In recent years, CV-A6 is re-emerging as the predominant causative agent of epidemics of HFMD worldwide, reported in Finland, Cuba, Argentina, Singapore and China [5–9]. A molecular epidemiological investigation of HFMD conducted by us in Xiangyang, China, revealed that a remarkably high proportion of HFMD cases were caused by CV-A6 with proportions of 59.54% [10]. Interestingly, CV-A6 has been found to be associated with adult HFMD [11]. Unlike the “classical” HFMD-associated enteroviruses, CV-A6 infection can lead to many atypical clinical symptoms such as vesiculobullous eruption, onychomadesis or herpangina. In addition, CV-A6 can cause serious central nervous system (CNS) disorders such as aseptic meningitis and brainstem encephalitis [12]. Recombination and spontaneous mutations have been addressed for effects on viral virulence of EV-A71 [13]. The reports of CV-A6 also demonstrated that mutations of the structural and nonstructural proteins may be responsible for the severity of the disease caused by CV-A6 [14,15]. Moreover, CV-A6 cocirculates with other enteroviruses, increases possibility of coinfection and produces new recombinant CV-A6 lineages attributing to the emerging of variants for recent outbreaks of CV-A6 [16,17].

Due to the increased prevalence and severity of CV-A6 infection, it is now recognized that CV-A6 should also be target for multivalent vaccine development to ensure a broad and effective protection against HFMD. Although highly effective vaccines have been commercially available in China for EV-A71 [18], the development of CV-A6 vaccines is challenging. For example, CV-A6 could not be efficiently isolated and propagate in Vero and MRC-5 cell lines, which are the two common cell substrates allowed for inactivated vaccine production used among infant and young children in China [19,20]. There is also a lack of adult, small animal models to perform active immunization-challenge to evaluate the efficacy of HFMD vaccines, although neonatal mouse models of EV-A71, CV-A16, CV-A10 and CV-A6 have been established [20–35].

As an alternative vaccine approach, recombinant virus-like particles (VLPs) of CV-A6 have been produced in a baculovirus/insect cell expression system [32]. Zhang et al. also combined CV-A6 VLP with VLPs derived from EV-A71, CV-A16 and CV-A10 to generate a VLP-based tetravalent vaccine [36]. In addition, the CV-A6 in RD cell cultures have been used to develop an inactivated bivalent and trivalent vaccine by Zhang and Caine, respectively [34,37]. However, RD cell line is not allowed to be used in human vaccine production.

In the present study, we report, to our knowledge, the first development of a Vero cell-based inactivated CV-A6 vaccine against HFMD. To evaluate the efficacy of CV-A6 vaccine, an actively immunized-challenged mouse model is established using a non-mouse-adapted challenge strain, which is lethal for 14-day-old Kunming mice at a fatality rate of 100%. This model demonstrates that inactivated, Vero-based CV-A6 vaccines completely protect 14-day-old Kunming mice against disease caused by CV-A6. The work will help to understand CV-A6 pathogenesis and the promising CV-A6 vaccine candidate could be used as one of the components in a multivalent vaccine against HFMD.

## Materials and methods

### Ethics statement

Mice and the procedures used for this study were approved by the Animal Ethics Committee of the Wuhan Institute of Biological Products (WIBP) (WIBP-AII 382020003), following the strict compliance with requirements of the Animal Ethics Procedures and Guidelines of the People’s Republic of China [38].

### Cells and viruses

Human rhabdomyoma (RD) cells and African green monkey kidney (Vero) cells were cultured in minimal essential medium (MEM, Nissui, Japan) and Dulbecco’s modified Eagle medium (DMEM, Thermo Fisher Scientific, USA), respectively. RD cells were cultured in MEM medium supplemented with 10% newborn bovine serum (NBS, Gibco), 2 mM glutamine, 100 U of penicillin and 100 μg of streptomycin per ml at 37°C in the presence of 5% CO_2_. A CV-A6 clinical isolate, CV-A6-HEV69/XY/CHN/2017 (GenBank ID MW410845), was isolated from specimens of HFMD patients in Xiangyang, China in 2017 [10]. The virus was passaged in RD cells for 8 times and purified by three consecutive end-dilution methods to obtain 19 clones. Two clones (CV-A6-R5 and CV-A6-R10) were selected to compare their virulence in Kunming mice. All virus harvests were subjected to three freeze–thaw cycles, clarified by centrifugation at 3900 × *g* for 10 min at 4°C and stored at −80°C. Titers of CV-A6 stocks were determined by 50% of cell culture infective dose (CCID_50_) assay with the method of Reed and Muench [39].

### Preparation of the inactivated CV-A6 vaccine

Another CV-A6 strain was isolated from specimens of HFMD patients in Xiangyang, China in 2018. The virus was first isolated in RD cells and then adapted to Vero cells. The full genome of Vero-adapted CV-A6 virus was sequenced and infectious cDNA was constructed to rescue Vero-adapted strains. Briefly, the developed processes of the vaccine preparation included the Vero cell culture and virus propagation, harvesting, followed by cell debris clarification, microfiltration, ultrafiltration, gel filtration chromatography, ion exchange chromatography and formaldehyde inactivation. The protein concentration of the vaccine stock was detected by enzyme-linked immunosorbent assay (ELISA) and formulation with buffer and Alum was performed.

### Mouse infection experiments

Five- and ten-day-old Kunming mice (n = 10 per group) were inoculated intracerebrally (i.c.) with CV-A6-R5 or CV-A6-R10 at a dose of 7.6 × 10^4^ CCID_50_/mouse and 1.1 × 10^5^ CCID_50_/mouse, respectively. Twelve-day-old Kunming mice (n = 6 per group) were employed in the experiments via i.c. and intraperitoneal (i.p.) inoculations with 1.7 × 10^7^ CCID_50_/mouse and 1.7 × 10^8^ CCID_50_/mouse of CV-A6-R10, respectively. The LD_50_ was determined in 14-day-old mice through i.p. route with CV-A6-R10 at doses of ten-fold serial dilutions of a highest dose (1.7 × 10^8^ CCID_50_/mouse). Uninfected control mice were administered with culture medium and kept in a separate cage from the infected mice. Mice were observed daily for clinical symptoms, body weight changes and fatality until 14 days post-infection (dpi). The grade of clinical symptoms was scored as follows: 0, healthy; 1, ruffled hair and hunchbacked; 2, limb weakness; 3, single limb paralysis; 4, double limb paralysis; 5, death [21]. The LD_50_ was calculated by using the Reed and Muench formula method [39], and the dose of 3.46 × 10^7^ CCID_50_/mouse (35 LD_50_) was optimized as the challenge dose in the following study.

### Active immunization/protection assay

Groups of Kunming mice (n = 10 per group) at days 3 and 9 after birth were i.p. primed and boosted with 1.5 µg or 4.5 µg in 100 µl of the purified and formaldehyde-inactivated CV-A6 vaccines. The antigens were formulated with adjuvants Aluminum hydroxide (Alum) from WIBP at 0.105 mg per dose. Another group of mice was injected with Alum-only and served as control. Five days after boosting, all mice were challenged i.p. with 3.46 × 10^7^ CCID_50_/mouse of CV-A6-R10, and then monitored daily for survival, body weight changes and clinical scores for 14 days. Mice were euthanized and blood or organ samples from experimental groups (n = 5) were collected at 0, 3 and 14 dpi. Tissues and organs of each group were collected, weighed and stored at −20°C for virus detection by quantitative real-time PCR (qRT-PCR). The organ samples used for histopathological examination and immunohistochemistry assay were collected separately and fixed with 4% paraformaldehyde.

### Neutralizing antibody (NtAb) assay

Neutralization assay was performed as reported by Shen [29] with small modifications. Antiserum collected from active immunization and control animals were inactivated at 56°C for 30 min before use. The serum samples collected from each mouse were 2-fold diluted (starting from dilution of 1:8) using MEM containing 5% FBS. Then, 50 µl of diluted serum was mixed with 50 µl (100 CCID_50_) of CV-A6 virus in 96-well plates in two duplicates and incubated at 37°C for 2 h. Cell and serum controls were included and virus back-titration was performed in a new 96-well plate. To do the virus back-titration, 50 μl of CV-A6 virus was 10-fold diluted (from 100 CCID_50_ to 0.1 CCID_50_) in eight duplicates for each dilution and mixed with 50 µl of MEM (free of FBS). Then, 1 × 10^4^ RD cells (100 µl) were added to each well of the 96-well plates. Seven days later, the cells were observed under a microscope for presence of cytopathic effects (CPE). Virus titers calculated from virus back-titration experiment were in the range of 32–320 CCID_50_/50 µl. The neutralizing titers were determined as the highest serum dilution at which CPE in 50% of the wells was completely inhibited.

### Quantitative real-Time PCR

The copy numbers of viral RNA were determined from collected organs of mice by qRT-PCR using in vitro-transcribed RNA standards. RNA standards (10^3^–10^10^ copies) were transcribed from the CV-A6 VP1 gene cloned into a plasmid pGEM-T easy vector (Promega, USA). The hydrolysis probe was labelled with a fluorescent dye (FAM) and a nonfluorescent black hole quencher (BHQ_2_) at the 5′- and 3′-ends, respectively. The forward primer VP1 F (5′-ATATTCGCAAAATTGAGTGATCCAC), reverse primer VP1 R (5′-GTTATTAGGACATTGCCCATATTGC) and hydrolysis VP1 probe (5′-FAM-ATCTGTCCCGTTCATGTCGCCAGC-BHQ_2_) were used to amplify a 150 bp fragment of the VP1 region. Viral RNA was extracted from 140 µl of tissue homogenates grinding with 1 g/10 ml MEM medium, using QIAamp viral RNA mini kit (Qiagen, Germany). The RNA sample was detected by one-step RT–PCR kit (Takara, Japan) in a reaction volume of 20 µl. Real-time PCRs were carried out for 40 cycles of 95°C for 5 s and 60°C for 40 s on the Applied Biosystems 7500 Fast Real-Time PCR System (Thermo fisher scientific, USA).

### Histopathologic and immunohistochemistry (IHC) analyses

Hematoxylin and eosin (HE) and IHC staining were analyzed by the Biofavor biotech corporation (China). Briefly, the tested tissues were separated, fixed, dehydrated, permeabilized and embedded in paraffin, which was then sliced into 4 µm sections. After staining with HE, sealed slides were examined by microscope.

For the immunohistochemistry detection, the tissue sections were dewaxed, dehydrated, and boiled for antigen retrieval in citrate buffer for 15 min. Rabbit anti-CV-A6 whole virus antiserum at a dilution of 1:200 was added for 15 h at 4°C. After primary antibody incubation, CV-A6 antigen was detected using a goat-anti-rabbit secondary antibody and a DAB peroxidase (HRP) substrate kit (Solarbio, China). After washing with PBS and dehydration, sealed slides were examined under a microscope.

### Cytokine assays

Levels of the interleukin-4 (IL-4), interleukin-6 (IL-6), tumour necrosis factor α (TNF-α) and interferon γ (IFN-γ) in serum of mice at 3 dpi were determined with individual mouse ELISA detection kits (Multi sciences Biotechnology Co., Ltd., China). The results are presented as mean values derived from duplicate tests. The theoretical limits of detection were as follows: IL-4, 0.08 pg/ml; IL-6, 0.43 pg/ml; TNF-α, 0.32 pg/ml; IFN-γ, 0.39 pg/ml.

### Statistical analyses

All statistical analyses were performed using GraphPad Prism 8 software (GraphPad Software Inc., USA). One-way analysis of variance (ANOVA) with Dunnett's multiple comparisons test were used. Statistical significance was indicated as follows: n.s., not significant (*P* ≥ 0.05); *, 0.01 ≤ *P* < 0.05; **, *P* < 0.01; ***, *P* < 0.001 and ****, *P* < 0.0001.

## Results

### Comparison of the virulence of different CV-A6 strains in mice

Nineteen clones were purified from a CV-A6 clinical isolate in RD cells and divided into nine genotypes based on their genome sequences. The experiment of one-step-growth curves was performed to characterize the growth ability of variants (data not shown). The CV-A6-R10 and CV-A6-R5 were selected to evaluate their virulence in Kunming mice based on their high and low titers in RD cells.

**Figure 1.**
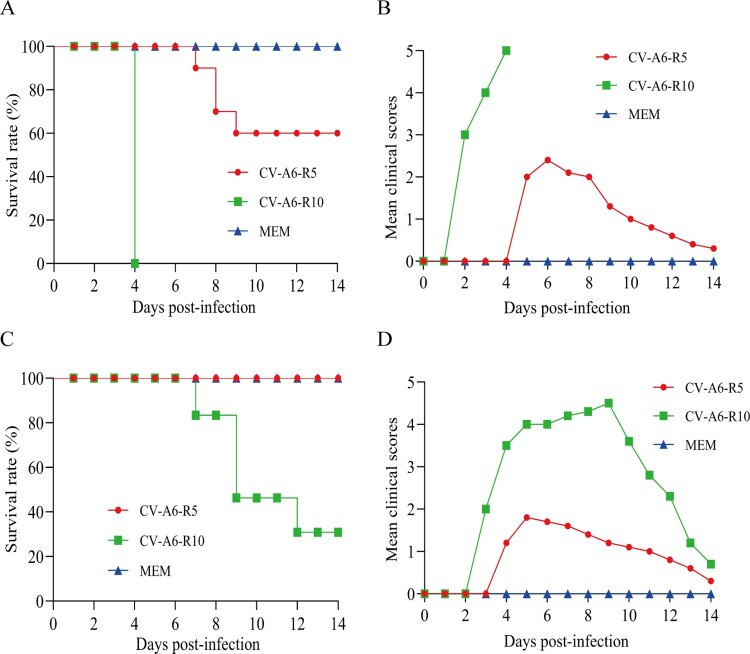
Virulence of CV-A6 in Kunming mice. Five-day-old (upper panels, 7.6 × 10^4^ CCID_50_/mouse) and ten-day-old (bottom panels, 1.1 × 10^5^ CCID_50_/mouse) Kunming mice (*n* = 10) were i.c. inoculated with CV-A6-R5 or CV-A6-R10. Control animals were inoculated with MEM medium. Survival rate (A and C) and mean clinical scores (B and D) were monitored and recorded daily after infection.

To compare the virulence of CV-A6-R5 and CV-A6-R10, groups of 5- or 10-day-old Kunming mice were inoculated intracerebrally with 7.6 × 10^4^ CCID_50_/mouse and 1.1 × 10^5^ CCID_50_/mouse, respectively. Clinical symptoms and survival rate were monitored daily after infection. As shown in Figure 1(A,B), 5-day-old mice infected with CV-A6-R10 exhibited limb paralysis and all died at 4 dpi. Whereas CV-A6-R5 infected mice developed clinical symptom, including limb weakness and paralysis, and 40% of them eventually died within 9 dpi. The survival rate of 10-day-old mice infected with CV-A6-R10 and CV-A6-R5 accounts for 30% and 100%, respectively (Figure 1(C)). The CV-A6-R5 infected group showed lower clinical scales than CV-A6-R10 infected group and turned to be healthy ultimately (Figure 1(D)). In contrast, no clinical symptoms were observed in the MEM-inoculated mice. These results indicated that CV-A6-R10 was much more virulent for Kunming mice than CV-A6-R5 and the virulence for mice was age- and dose-dependent. Therefore, CV-A6-R10 was selected as the challenge strain for further characterization.

The full-length nucleotide sequences of the two challenge strains were compared. Two nucleotide changes were identified and caused two amino acid substitutions at positions 124 and 242 of the VP1, from V to M and I to V, respectively. These two mutations may be associated with the virulence differences between CV-A6-R5 and CV-A6-R10.

### Establishment of a lethal mouse model for active immunization and challenge

**Figure 2.**
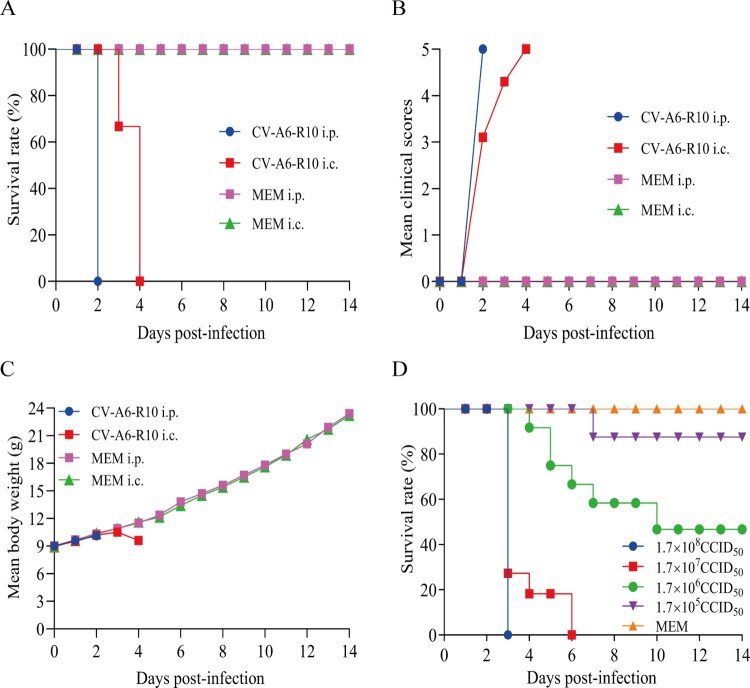
Determination of the optimal infection route and establishment of the Kunming mouse model. Twelve-day-old mice (*n* = 6) were i.p. and i.c. inoculated with CV-A6-R10 at doses of 1.7 × 10^8^ CCID_50_/mouse and 1.7 × 10^7^ CCID_50_/mouse, respectively. Control animals were inoculated with MEM medium. All the mice were monitored daily for survival rate (A), mean clinical scores (B) and mean body weight (C) until 14 days post-infection (dpi). The LD_50_ of CV-A6-R10 was determined through the i.p. route at the doses indicated (D).

To further investigate dose effect and assess the effect of the inoculation routes on morbility and mortality, 12-day-old Kunming mice were i.p. or i.c. infected with CV-A6-R10 at doses of 1.7 × 10^8^ CCID_50_/mouse and 1.7 × 10^7^ CCID_50_/mouse, respectively. The survival rate, body weight and clinical scores were monitored for 14 dpi (Figure 2(A–C)). The results showed that inoculation via the i.p. route resulted in severe clinical signs, such as weakness and paralysis, and the mortality rate was 100% at 2 dpi. Mice i.c. inoculated began to show symptoms at 2 dpi and eventually all died within 4 dpi, two days later compared with i.p. challenged group. In contrast, 100% of MEM-inoculated mice survived for 14 dpi. These observations suggested that the i.p. route was a more effective route of infection than the i.c. route mainly due to 10-fold higher dose inoculation (large volume of virus inoculation allowed). Moreover, the i.p. infection is easier to perform, which can enhance the reliability of the experiments. Thus, the i.p. route was used in the following experiments.

To examine the correlation between the infective dose and survival rate, 14-day-old Kunming mice were i.p. challenged with 10-fold serially diluted CV-A6-R10. Mice infected with the doses of 1.7 × 10^7^ and 1.7 × 10^8^ CCID_50_/mouse started to die at 3 dpi, and all were dead within 6 and 3 dpi, respectively. With the inoculated doses of 1.7 × 10^5^ and 1.7 × 10^6^ CCID_50_/mouse, mice began to show symptoms at 7 and 4 dpi, and the survival rates were 90% and 50%, respectively (Figure 2(D)). In contrast, no clinical incidents were observed in MEM-inoculated mice. Therefore, the onset time of symptoms and survival rates had a good correlation with the infective dose, and the LD_50_ was calculated as 1 × 10^6^ CCID_50_/mouse. Moreover, the clinical symptoms of the mice infected with 1.7 × 10^8^ and 1.7 × 10^7^ CCID_50_/mouse started to be exhibited mean clinical scores of grades 5 (death) and 3 (single limb paralysis) within 3 dpi, respectively. The symptoms of all mice were graded 5 within 6 dpi (data not shown). The results demonstrated that CV-A6-R10 could develop severe symptoms and lead to death in 14-day-old mice if the mice were inoculated via i.p. route with a higher dose. Hence, a dose of 3.46 × 10^7^ CCID_50_/mouse (35 LD_50_) was chosen for subsequent active immunization-challenge experiments.

### Efficacy of the CV-A6 vaccine candidate in Kunming mice

**Figure 3.**
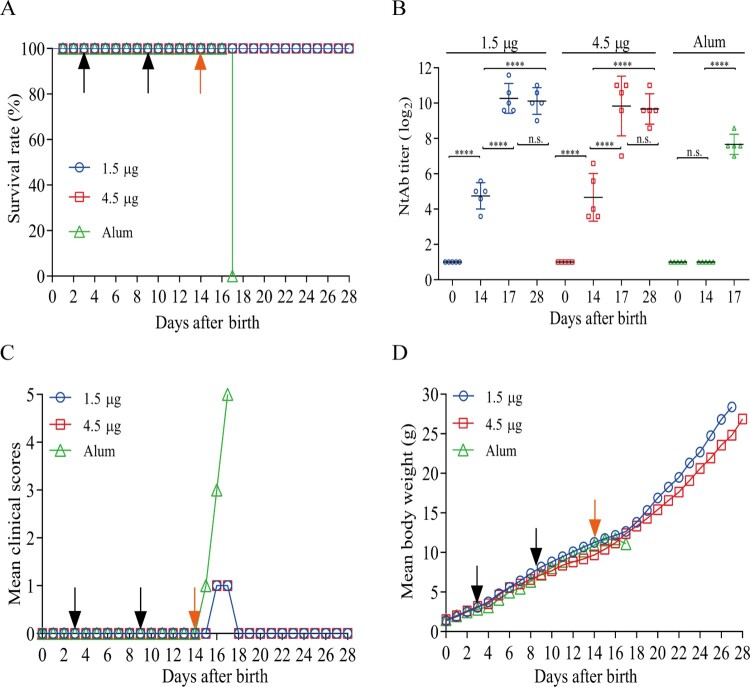
Efficacy of the vaccine in mice immunized with the two vaccine formulations. Immunization was performed in groups of 14-day-old Kunming mice (n = 10) via i.p. route. High and low doses (4.5 μg and 1.5 μg/mouse) of the inactivated vaccine candidate were inoculated with adjuvants Alum. Control group were inoculated with Alum-only. Kunming mice were primed and boosted on days 3 and 9 and challenged on day 14 at a dose of 35 LD_50_ (3.46 × 10^7^ CCID_50_/mouse). Bleeding was performed on days 0, 14, 17 and 28. Black and orange arrowheads indicated the date of vaccination and challenge, respectively. The inoculated mice were monitored for survival rate (A), mean clinical scores (C) and mean body weight (D) for a period of 14 days. (B) NtAb titers of serum of mice vaccinated with vaccines or Alum-only (n = 5) were determined and presented as the geometric mean titer (GMT) ± the standard error of the mean (SEM). NtAb titers below 8 were assigned to 2 for convenience of figure presentation. Each symbol represented a mouse, and the solid line indicated the GMT of the group. The data were analyzed with one-way ANOVA. **** and n.s. indicating P < 0.0001 and no significant difference (*P* ≥ 0.05), respectively.

The efficacy of the inactivated CV-A6 vaccine was evaluated by the active immunization of Kunming mice, followed by challenging with CV-A6-R10 through the i.p. route. Groups of 1-day-old mice were primed and boosted with 1.5 and 4.5 µg of the inactivated CV-A6 vaccine or the Alum-only at days 3 and 9. The mice were then challenged with CV-A6-R10 at a dose of 35 LD_50_ (3.46 × 10^7^ CCID_50_/mouse) five days later when antibody levels had increased. The mice that had received the inactivated vaccine exhibited very minor symptoms and were completely protected from death during the 14-day observation period. In contrast, the mice given the Alum-only quickly developed clinical manifestations including limb weakness and paralysis after CV-A6-R10 challenge, and all of them died at 3 dpi (Figure 3(A)).

Levels of NtAb titers in serum on days 0, 14, 17 and 28 were determined, representing levels at time points of pre-immunization, post-boost, postchallenge and end of observation period (Figure 3(B)). The data showed that NtAbs were undetectable at day 0 and could be induced in mice after boosting at day 14. The serum conversion rate was 100%. Following challenge with CV-A6-R10, a strong immune response was induced. Seventeen-day-old mice immunized with two doses of 1.5 µg or 4.5 µg vaccines exhibited a high NtAb titer (1,230 or 911, respectively), compared with that of the control group administered with Alum-only (202). Importantly, the 100% of seroconversion of NtAb correlated with the complete survival of all immunized mice. No obvious differences in NtAb titers were observed between days 17 and 28 in each group of the vaccinated-immunized mice. As shown in Figure 3(C,D), at the early stage before challenge, the immunized and Alum-only control groups had the similar trend in clinical scores and body weight changes, indicating tolerance of mice to the vaccine. However, body weight dropped and clinical scores increased sharply for Alum-only inoculated mice after challenge, compared with those for mice in the two immunized groups. These results demonstrated that active immunization with the inactivated CV-A6 vaccine was able to confer full protection from the disease caused by CV-A6.

### Viral loads in different organs of CV-A6 immunized-challenged mice

**Figure 4.**
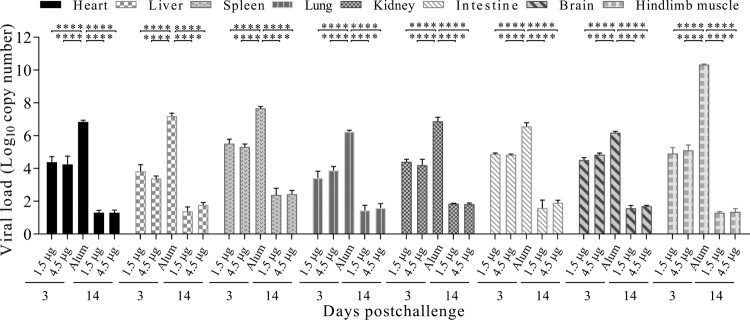
Virus distributions in various organs of immunized-challenged Kunming mice. Kunming mice immunized with 1.5 μg, 4.5 μg vaccines or Alum-only were i.p. inoculated with 35 LD_50_ (3.46 × 10^7^ CCID_50_/mouse) of CV-A6-R10. The graph showed the virus loads in the heart, liver, spleen, lung, kidney, intestine, brain and hindlimb muscle tissues at 3 dpi or 14 dpi. Virus loads were assessed by qRT-PCR and compared with standard curves obtained from 10-fold serial dilutions of CV-A6 transcript. The data represent the mean ± SEM for five mice per group and were analyzed with one-way ANOVA (****, *P* < 0.0001).

To further understand the invade and spread route of CV-A6-R10 in the immunized-challenged mice, the viral loads in different organs were detected by qRT-PCR. As shown in Figure 4, 3 days after challenge, the viral loads in all organs of immunized-challenged mice were lower than those of Alum-only inoculated mice (*P* < 0.0001). The viral loads of the examined organs decreased in the order of muscle, spleen, liver, kidney, heart, intestine, lung and brain. Furthermore, at the end of observation period (14 dpi), the viral loads dropped strikingly in the immunized-challenged groups. There were no significant differences between the two vaccine formulations at 3 dpi or 14 dpi. These findings indicated that the hindlimb muscle was the major site of viral propagation. Detection of viral RNA in brain indicated invading of CV-A6 into CNS.

### Histopathologic and immunohistochemistry analyses

**Figure 5.**
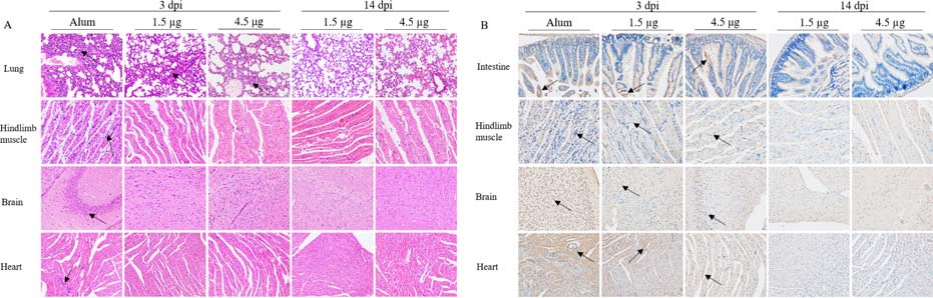
Histopathologic and IHC analyses of tissues from immunized-challenged Kunming mice. Histological (A) and IHC (B) of various tissues from immunized-challenged Kunming mice were analyzed. Immunized Kunming mice were i.p. inoculated with a lethal dose (3.46 × 10^7^ CCID50/mouse) of CV-A6-R10. The Alum-only groups were euthanized immediately following death on 3 dpi, and mice in the vaccinated groups were euthanized at 3 dpi and 14 dpi. Sections from lung, muscle, brain and heart tissues were stained with hematoxylin to detect pathological changes. Viral proteins were detected in IHC assays with an anti-CV-A6 whole virus polyclonal antibody as the primary antibody. Original magnification, × 200. Images shown are representative of two Kunming mice in each group. Black arrowheads indicated representative inflammatory cell infiltration (A) and expression of viral antigen (B).

HE and IHC experiments were performed at days 3 and 14 after CV-A6-R10 challenge to compare the pathological changes and antigenic distribution in tissues of the immunized-challenged Kunming mice or control mice. The results showed that the virus had strong tropism to the hindlimb muscle and lung tissues in the Alum-only inoculated mice (Figure 5(A)). Viral propagation was associated with severe pathological damage, accompanied by large numbers of muscle bundle fracture and fibrosis. The lungs showed interstitial fibrosis and inflammatory hyperemia. Severe damage including necrosis and increased numbers of inflammatory cells were shown in the brain and heart tissue. In the IHC experiments, the widespread CV-A6 viral proteins was detected in the intestine, hindlimb muscle, brain and heart tissues of the Alum-only inoculated mice (Figure 5(B)).

Although the lung from 1.5 and 4.5 µg vaccinated-challenged mice exhibited severe pathological features at 3 dpi, no obvious pathological changes were observed in the two vaccinated groups at 14 dpi. Other tissues of immunized-challenged mice showed the similar trend in the pathological changes at 3 and 14 dpi (Figure 5(A)). The levels of viral protein expression in the intestines, brains, hindlimb muscles and hearts of immunized-challenged mice were lower than those in the Alum-only inoculated mice at 3 dpi, and no obvious viral protein was detected at 14 dpi (Figure 5(B)). The results of HE and IHC demonstrated that the inactivated vaccine protected the mice from disease with only slight and quickly-recovered infection and caused no significant pathological damage in the mice.

### Cytokine levels in the serum of CV-A6 immunized-challenged mice at 3 dpi

**Figure 6.**
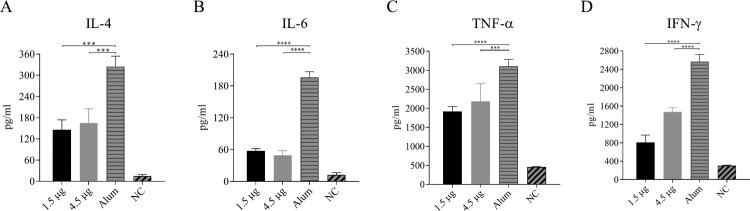
Expression of inflammatory cytokines in serum from immunized-challenged Kunming mice. Immunized (vaccine or Alum-only) mice were i.p. inoculated with a lethal dose (3.46 × 10^7^ CCID_50_/mouse) of CV-A6-R10. The levels of IL-4 (A), IL-6 (B), TNF-α (C) and IFN-γ (D) in the serum of Kunming mice were determined with individual mouse ELISA detection kits at 3 dpi. Data represent the mean results of three experiments ± SEM and were analyzed with one-way ANOVA (****, *P* < 0.0001; ***, *P* < 0.001). NC, PBS-inoculated negative control.

Enhanced cytokine production has been proposed to contribute to EV-A71 pathogenesis in both humans and mice [40–44]. It is worth to investigate whether the similar phenomenon exist in CV-A6 infected mice. The expression of inflammatory cytokines in the serum was measured at 3 dpi after challenge with 35 LD_50_ of CV-A6-R10 following the priming and boosting with 1.5, 4.5 µg of inactivated vaccines or Alum-only. The expression levels of the IL-4, IL-6, TNF-α and IFN-γ in the serum of Alum-only inoculated group were significantly higher than those in the two vaccinated groups and PBS-inoculated negative control (NC) (Figure 6). Importantly, the expression levels of IL-4, IL-6 and IFN-γ in Alum-only inoculated group showed an extremely sharp increase at 324, 195 pg/ml and 2,564 pg/ml, respectively. These results suggested that IL-4, IL-6, IFN-γ and TNF-α might be the major factors in the inflammatory responses during CV-A6 infection in Kunming mice.

## Discussion

Establishment of animal models of enteroviruses is critical for antiviral drug screening, vaccine development and pathogenesis studies. For EV-A71, there have been numerous attempts to develop animal models. Strategies have included using clinical isolates [21,22] or mouse adaptation strains [23,24], and using immunodeficient mice [21] as well as receptor-transgenic mice [26,27]. Mouse models have also been reported for other enteroviruses associated with HFMD, such as CV-A16, CV-A6, CV-A10, CV-A4 and CV-A5 [28–37,45,46]. However, the challenging viruses used infected suckling mice younger than 3–5 days in most reports. Therefore, the conventional way to evaluate the efficacy of vaccine candidates is passively immunized mouse model [3,47,48]. This passive immunization model does not mimic the cellular and humoral responses directly induced by vaccines in neonatal mice. Because of the upsurge in epidemic HFMD attributable to CV-A6 in recent years, a mouse model suitable for evaluating active immunity for CV-A6 is urgently needed.

In our previous study, we reported an active immunization-challenge model for CV-A5 established by using a mouse-adapted strain, CV-A5-M14 [46]. Unlike CV-A5 model, non-mouse-adapted CV-A6 virus was used to develop an active immunization model in this study. Compared with mouse-adapted challenge strains, this method is time saving and could avoid changing the natural tropism of the virus via adaptation in the mouse brain. The homology of non-mouse-adapted CV-A6 virus is also close to vaccine candidate strains. In this study, virus growth ability was enhanced in RD cells for eight passages and virus plaque-purifications were carried out. The obtained 19 clones displayed different growth abilities in RD cells. Two strains were selected, abbreviated as CV-A6-R10 and CV-A6-R5, showing different virulence in Kunming mice. Two substitutions in amino acid sequences (V124M and I242 V) in VP1 region of these two clones may be associated with the virulence of CV-A6. The mutations may be generated by consecutive passages in RD cells or originated from the specimen of the HFMD patient, which might be a quasispecies. The term “quasispecies” was first adopted and further developed by Domingo to describe a population that are complex, dynamic distributions of nonidentical but related genomes [49,50]. This phenomenon occurred both in natural evolution and cell culture. A VP1 mutation (K244E) in a mouse-adapted EV-A71 was previously shown to be necessary for EV-A71 virulence in adult AG129 mice [51]. The I242 V mutation in the VP1 of CV-A6 was speculated to have a similar function due to the close distance from K244, although lacking of structural research. Further work is required to study mechanism of CV-A6 virulence using avirulent clones from the same virus stock.

In previous research, EV-A71 and CV-A16 infection not only had a muscle tropism but also entered the brain and spinal cord, resulting in CNS complications, reported by Wang et al. and Mao et al., respectively [23,28]. Similarly, a CV-A6 and CV-A10 infection model showed that CV-A6 and A10 had a strong tropism to muscle [30,35]. In this study, histopathological analysis and tissue viral RNA detection showed that CV-A6 had a stronger tropism to muscle than other organs in Kunming mice, indicating that muscle was the most active site of viral proliferation. This could be one of the direct causes of limb paralysis in neonatal mice. The intestine (the primary infection site of enteroviruses) and cardiac tissues were also the sites of CV-A6 accumulation based on IHC and viral load results. Furthermore, the appearance of viruses in the brain, as well as the relatively high viral load suggested that CV-A6 had successfully entered the CNS of Kunming mice. One route of entering the CNS used by poliovirus is most likely through retrograde axonal transport, a pathway the virus trafficked from the neurons of the peripheral nervous system to the CNS [52]. The pathological feature was similar to that previously reported for CV-A6 [33] and differed from the reports of CV-A10 animal models [30], which might be attributable to the different serotypes.

Taken together, our data indicated that after intraperitoneal injection, CV-A6-R10 was replicated in muscle and arrived in target tissues through blood circulation. The viruses spread to the whole body, then caused lesions and necrosis in cardiac muscles and finally led to death. This was in agreement with previous report of poliovirus, speculating that skeletal muscle has been proposed to support persistent enterovirus infection and to represent a viral source of entry into the CNS during poliovirus infection [53]. Obviously, further work needs to be performed through a natural, oral route infection in this mouse model.

The role of viral versus immunological factors in EV-A71 pathogenesis has been extensively investigated in previous studies. Upon invasion by enteroviruses, inflammatory mediators are generated in “signaling cascades” through a series of pathways and result in systemic inflammatory response syndrome. However, overexpression or imbalance in the expression of these immune factors can aggravate the inflammatory response to infection, causing damage to infected tissues and leading to disorders of organ function or even death [42]. Some researchers proposed that sustained high levels of IL-6 alone could cause severe tissue damage [40]. Duan et al. reported that the IL-4, IL-6, interleukin-10 (IL-10), TNF-α and IFN-γ levels correlated with the progress of HFMD. The levels of IL-4, IL-6, IL-10 and IFN-γ during the progression to severe HFMD significantly increased from the 2nd day to 4th day and then decreased. The levels of TNF-α were high on the first and second day and then significantly decreased [44].

In our present study, the expression levels of IL-4, IL-6, TNF-α and IFN-γ in Alum-only inoculated group (grade 4-5) at 3 dpi were remarkably higher than the vaccinated-challenged group. This phenomenon could be explained for the rapid progression to severe symptom of CV-A6, resulting in overexpressing a high imbalanced level of anti- and pro-inflammatory cytokines to clear virus-infected cells. A long-term persistence of immunity and memory were established by priming and boosting with the inactivated vaccine, which rendered a swift immune response when the CV-A6 antigens were re-recognized after challenge. Whether the inactivated CV-A6 vaccine candidate could elicit T-cell response in mice should be further investigated by other approach, such as enzyme-linked immunospot (ELISPOT) assay which estimated the specific CD4^+^ or CD8^+^ T cell proliferation restimulating with specific peptides derived from viral structural proteins.

In conclusion, a end-dilution-purified, virulent cell isolate was used as a challenge strain to develop a CV-A6 active immunization mouse model for evaluation of the efficacy of vaccines. The inactivated CV-A6 vaccines in different doses were capable of conferring complete protection against lethal CV-A6-R10 challenge in the 14-day-old mouse. The results revealed that CV-A6-R10 had a strong tropism for limb muscle tissues, invading CNS and causing severe necrosis and paralysis. The data indicated that IL-4, IL-6, TNF-α and IFN-γ might be associated with pathogenesis. The mouse model described here will be a useful tool to provide a more comprehensive understanding of HFMD, which in turn, will be helpful in the development of safe and effective multivalent vaccines against HFMD.
